# Basic Characterization of Natural Transformation in Avibacterium paragallinarum

**DOI:** 10.1128/spectrum.05209-22

**Published:** 2023-05-22

**Authors:** Donghui Liu, Hao Zhang, Huihui Tan, Yikun Jin, Chengcheng Zhang, Zongyi Bo, Xiaorong Zhang, Mengjiao Guo, Yantao Wu

**Affiliations:** a Jiangsu Co-Innovation Center for Prevention of Animal Infectious Diseases and Zoonoses, College of Veterinary Medicine, Yangzhou University, Yangzhou, Jiangsu, China; b Joint International Research Laboratory of Agriculture and Agri-Product Safety, Yangzhou University (JIRLAAPS), Yangzhou, Jiangsu, China; University of Minnesota—Twin Cities

**Keywords:** natural transformation, *Avibacterium paragallinarum*, uptake signal sequence, infectious coryza

## Abstract

Avibacterium paragallinarum is the pathogen involved in infectious coryza (IC), an acute infectious upper respiratory disease in chickens. The prevalence of IC has increased in China in recent years. There is a lack of reliable and effective procedures for gene manipulation, which has limited the research on the bacterial genetics and pathogenesis of A. paragallinarum. Natural transformation has been developed as a method of gene manipulation in *Pasteurellaceae* by the introduction of foreign genes or DNA fragments into bacterial cells, but there has been no report on natural transformation in *A. paragallinarum*. In this study, we analyzed the existence of homologous genetic factors and competence proteins underlying natural transformation in *A. paragallinarum* and established a method for transformation in it. Through bioinformatics analysis, we identified 16 homologs of Haemophilus influenzae competence proteins in *A. paragallinarum*. We found that the uptake signal sequence (USS) was overrepresented in the genome of *A. paragallinarum* (1,537 to 1,641 copies of the core sequence ACCGCACTT). We then constructed a plasmid, pEA-KU, that carries the USS and a plasmid, pEA-K, without the USS. These plasmids can be transferred via natural transformation into naturally competent strains of *A. paragallinarum*. Significantly, the plasmid that carries USS showed a higher transformation efficiency. In summary, our results demonstrate that *A. paragallinarum* has the ability to undergo natural transformation. These findings should prove to be a valuable tool for gene manipulation in *A. paragallinarum*.

**IMPORTANCE** Natural transformation is an important mechanism for bacteria to acquire exogenous DNA molecules during the process of evolution. Additionally, it can also be used as a method to introduce foreign genes into bacteria under laboratory conditions. Natural transformation does not require equipment such as an electroporation apparatus. It is easy to perform and is similar to gene transfer under natural conditions. However, there have been no reports on natural transformation in Avibacterium paragallinarum. In this study, we analyzed the presence of homologous genetic factors and competence proteins underlying natural transformation in *A. paragallinarum*. Our results indicate that natural competence could be induced in *A. paragallinarum* serovars A, B, and C. Furthermore, the method that we established to transform plasmids into naturally competent *A. paragallinarum* strains was stable and efficient.

## INTRODUCTION

Avibacterium paragallinarum belongs to the Gram-negative bacterial family *Pasteurellaceae*. It is the etiological agent of infectious coryza (IC), a severe acute respiratory disease in chickens (1). The pathogenicity mechanism of A. paragallinarum remains unclear, and research on its virulence factors is limited. One important reason for this is the lack of stable and effective genetic manipulation methods. The introduction of DNA into the cytoplasm is a prerequisite for genetic manipulation in bacteria. Although electroporation is a common and efficient method for transformation in bacteria (2), it is not always effective in *A. paragallinarum* (3, 4). However, some genera of the *Pasteurellaceae*, such as Haemophilus, *Pasteurella*, *Riemerella*, and others, are naturally competent (5–7). These genera actively take up DNA from their surroundings as nutrients or incorporate DNA into their genomes via homologous recombination (5, 8–10).

Competent bacteria have a series of competence proteins that allow them to accomplish natural transformation. In Haemophilus influenzae, competence is induced when nutrients become limited. Cells of H. influenzae acquire competence once they are transferred from a nutrient-rich medium to a starvation medium (11–13). In addition, two regulatory proteins, cyclic AMP (cAMP) receptor protein (CRP) and transcriptional coactivator for CRP (Sxy [also called TfoX in H. influenzae]), are required to induce competence in H. influenzae (14, 15).

The concentration of the signal molecule cAMP increases in the absence of nutrients, thus activating its receptor protein CRP and enabling the formation of the cAMP-CRP complex. When combined with Sxy, the activated CRP binds to a specific promoter sequence (CRP-S) that is common to the competence genes responsible for DNA uptake and processing. This binding leads to the induction of gene transcription by the recruitment of RNA polymerase (16, 17). Additionally, certain specific sequences may enhance the effectiveness of the natural transformation process. The cell surface’s DNA uptake mechanism has a preference for binding and ingesting fragments that contain the uptake signal sequence (USS) (18).

Natural transformation has been extensively employed for the generation of targeted mutants (18–22) and is a convenient tool for the study of bacterial genetics and pathogenesis. However, there is a lack of relevant research on *A. paragallinarum*. Therefore, the goals of this work were to analyze the homologous genetic factors and competence proteins underlying natural transformation in *A. paragallinarum* and to develop a method to induce its transformation.

## RESULTS

### Putative competence proteins and regulators identified in *A. paragallinarum*.

The analysis identified 16 competence proteins and regulators in *A. paragallinarum* genomes (Table 1). CRP and Sxy are responsible for the regulation of natural transformation, while the other 14 competence proteins are closely associated with DNA uptake (ComE, ComF, PilA, PilB, PilC, PilD, PulG, ComEC, and PilW) and DNA processing (ComM, DprA, RadC, Ssb, and LigA) (Table 2). The presence of homologous competence proteins and regulators indicated that *A. paragallinarum* may be naturally competent like H. influenzae. However, there are no homologs of some competence proteins in *A. paragallinarum* (such as ComA, ComB, ComC, and ComD). Further investigation is required to determine the reason for the lack of these proteins in *A. paragallinarum* and its influence.

**TABLE 1:** *A. paragallinarum* homologs of H. influenzae competence proteins^*a*^

Category and H. influenzae Rd KW20 protein	H. influenzae Rd KW20 protein length (aa)	*A. paragallinarum* ADL-AP02			*A. paragallinarum* 2019/JS28		
		Protein homolog	Protein length (aa)	Sequence ID (%)^*b*^	Protein homolog	Protein length (aa)	Sequence ID (%)^*c*^
Regulation							
CRP	224	CRP (HHJ57_01975)	203	79.8	JS28000144	203	99.5–100
Sxy	217	Sxy/TfoX (HHJ57_09260)	216	34.9	JS28002400	216	100
DNA uptake							
ComA	265	Absent			Absent		
ComB	168	Absent			Absent		
ComC	173	Absent			Absent		
ComD	137	Absent			Absent		
ComE	445	PilQ/ComE (HHJ57_01660)	440	64.6	JS28000092	440	100
ComF	229	ComF (HHJ57_10495)	230	58.0	JS28001982	168	98.7–100
ComE1	112	Absent			Absent		
PilA	149	PilA (HHJ57_07795)	143	60.1	JS28001547	143	100
PilB	464	PilB (HHJ57_07790)	474	66.1	JS28001548	474	99.8–100
PilC	406	PilC (HHJ57_07620)	409	52.2	JS28001581	404	99.5–100
PilD	230	PilD (HHJ57_07615)	231	36.8	JS28001582	231	97.4–100
ComN/PulG	170	PulG (HHJ57_08755)	173	51.2	JS28001936	173	98.8–100
ComO	238	Absent			Absent		
ComP	227	Absent			Absent		
ComQ	101	Absent			Absent		
Rec2	788	ComEC/Rec2 (HHJ57_06035)	821	53.4	JS28001226	830	98.2–100
PilF2	179	PilW/PilF2 (HHJ57_08580)	180	50.0	JS28000037	180	99.4–100
DNA processing							
ComM	509	ComM/YifB (HHJ57_09565)	511	82.5	JS28002238	500	99.8–100
DprA	373	DprA (HHJ57_08575)	366	62.3	JS28000038	361	99.7–100
RadC	234	RadC (HHJ57_02000)	218	67.4	JS28000149	218	100
Ssb	168	Ssb (HHJ57_01285)	159	65.4	JS28001425	158	80.4–100
LigA	185	LigA (HHJ57_01830)	675	27.6	JS28000113	675	99.3–100

a ID, identity; aa, amino acids.

b Sequence identity between predicted proteins from *A. paragallinarum* ADL-AP02 and H. influenzae Rd KW20.

c Sequence identity among *A. paragallinarum* strains.

**TABLE 2:** Competence proteins of *A. paragallinarum*

Protein	Description	Homolog(s) (organism[s])	Function(s) of homolog(s) (reference[s])
CRP	Cyclic AMP receptor protein	CRP (*Neisseria*, Haemophilus)	Global transcription regulator required for bacterial competence (16, 17)
Sxy	DNA transformation protein	Sxy (*Neisseria*, Haemophilus)	Induces natural DNA uptake by inducing the transcription of competence genes (CRP-S regulon) (16, 17)
PilQ/ComE	Type IV pilus biogenesis and competence protein	PilQ (*Neisseria*), ComE (Haemophilus)	Outer membrane secretin; binds the type IV pilus and DNA (23, 24)
ComF	Competence protein F	ComF (*Neisseria*, Haemophilus)	Function unknown
PilA	Type IV major pilin protein	PilE (*Neisseria*), PilA (Haemophilus)	Major pilin subunit (25)
PilB	Type IV pilus assembly ATPase	PilB (*Neisseria*, Haemophilus)	ATP-dependent extension of pilus subunits (26)
PilC	Type IV pilus assembly protein	PilC (*Neisseria*, Haemophilus)	Pilus assembly (26, 27)
PilD	Prepilin leader peptidase	PilD (*Neisseria*, Haemophilus)	Prepilin peptidase (26–28)
PulG/ComN	Type II secretion system core protein G	ComN (Haemophilus)	Function unknown
ComEC/Rec2	Recombination protein 2	Rec2 (Haemophilus), ComEC (*Bacillus*)	Putative DNA membrane channel (29)
PilW/PilF2	Type 4 fimbrial biogenesis protein	PilW (*Neisseria*), PilF (Haemophilus)	Pilotin; stabilizes secretin multimers (30–33)
ComM	Competence protein M	ComM (*Neisseria*, Haemophilus)	Function unknown
DprA	DNA-processing protein A	DprA (*Neisseria*, Haemophilus)	Protects DNA from degradation in the cytoplasm (34)
RadC	DNA repair and recombination protein	RadC (Haemophilus)	Replication fork stabilization and repair (35, 36)
Ssb	Single-stranded DNA-binding protein	Ssb (*Neisseria*, H. influenzae)	Ubiquitous single-stranded DNA-binding protein (37)
LigA	DNA ligase A	Adl (*Neisseria*), LigA (H. influenzae)	Periplasmic ATP-dependent DNA ligase (38)

The results of the sequence analysis demonstrated that the competence proteins among the *A. paragallinarum* strains exhibited >97% sequence identity, except for Ssb, whose sequence identity was >80.4% (Table 1). These findings clearly suggest that the proteins linked with natural transformation in *A. paragallinarum* are remarkably conserved.

**FIG 1:**
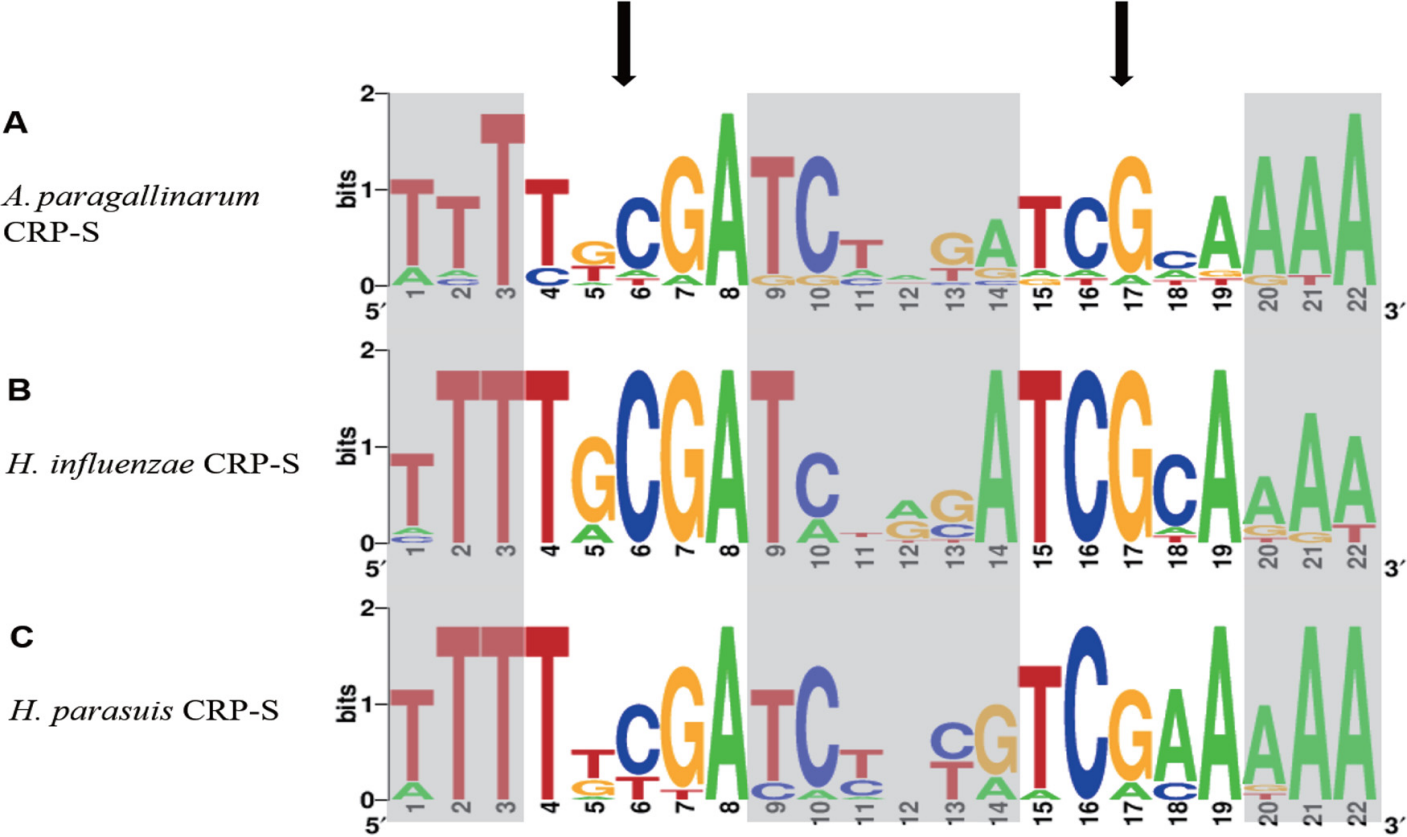
(A) Sequence logo for CRP-S sites of *A. paragallinarum* (based on 11 *A. paragallinarum* sites). (B) Sequence logo for CRP-S sites of H. influenzae (based on 11 H. influenzae sites [21]). (C) Sequence logo for CRP-S sites of H. parasuis (based on 12 H. parasuis sites [7]). The characteristic positions of CRP-S sites (C6/G17) are shown with arrows. Noncore sites are shown in gray shading.

In our study, an examination of the promoter regions of competence homologs in *A. paragallinarum* revealed elements resembling the CRP-S sites identified in other bacteria. We identified these consensus sequences in the upstream DNA sequences of 11 transcriptional units of competence homologs in *A. paragallinarum* (Table 3). These reverse-complement consensus sequences exhibit high levels of similarity to the CRP-S consensus sequences of H. influenzae and Haemophilus parasuis (Fig. 1).

**TABLE 3:** Predicted sequences of CRP-S sites in *A. paragallinarum*

Gene	*P* value	CRP-S site sequence (5′–3′)
*comF*	7.62e−08	TTTTGCGATCTAGATCGTAAAA
*comM*	2.57e−06	TCTCTCGATCTAGATCGCAAAA
*crp*	2.03e−05	TTTTTTAATCACTGATGATGAA
*dprA*	2.16e−09	TTTTGCGATCTTTCTCGCAAAA
*pilA*	1.02e−07	TTTTGCGATCCAGATCGAGAAA
*pilC*	5.86e−07	TATTGAGATGTGGATCGTAAAA
*pilQ*	6.97e−05	TTTCTCGATCAAGAGAAAAAAA
*pulG*	7.4e−09	TTTTGCGATCTGTATCGCAATA
*radC*	9.73e−09	ATTTTCGATCCTGATCGCAAAA
*rec2*	1.58e−07	TTTTGCGAGCTAGATCGCAAAA
*sxy*	4.12e−10	ATTTACGATCTACGTCGCAAAA

In H. influenzae, 17 genes regulated by CRP-S are necessary for natural transformation (23). These CRP-S sites have been identified as binding sites for CRP under Sxy regulation. Notably, the CRP-S sites differ from canonical CRP-N sites in that the former require both CRP and Sxy proteins for transcription activation and the 6th base is cytosine instead of thymine (7, 17).

Upon analyzing the genomes of the *A. paragallinarum* strains, it was discovered that there were 1,537 to 1,641 copies of the 9-bp short DNA sequence 5′-ACCGCACTT-3′ (Table 4). This high number suggests that this particular DNA sequence is enriched in the genome of *A. paragallinarum*. Conversely, the DNA sequence 5′-ACAAGCGGT-3′ was found to have only 38 to 45 copies in the genome, while the sequence 5′-ACCGAACTC-3′ had only 10 to 11 copies. Additionally, we compared the core USS of *Neisseria* (5′-GCCGTCTGAA-3′) and found that there were only 6 to 22 copies present in the genomes of the *A. paragallinarum* strains. It is worth noting that the genomes of bacteria with natural competence exhibit high levels of enrichment in their preferred sequences, meaning that one or more USSs would appear in any fragment larger than approximately 2 kb (5). Therefore, only the core DNA sequence of H. influenzae is consistent with the characteristics of the USS in *A. paragallinarum*.

**TABLE 4:** Analysis of the USSs of *A. paragallinarum*

Representative bacterium	USS (5′–3′)	No. of copies in the genome of *A. paragallinarum* strain:							
		2019/JS28	2019/JS40	2019/JS44	FARPER-174	ESV-135	p4chr1	ADL-AP02	AVPG2015
*Neisseria*	GCCGTCTGAA	19	17	22	6	12	19	11	12

H. influenzae	ACCGCACTT	1,622	1,555	1,610	1,537	1,592	1,641	1,560	1,596

H. parasuis	ACAAGCGGT	41	38	40	42	43	43	40	45
	ACCGAACTC	11	11	11	10	11	11	11	11

### Induction of natural competence in *A. paragallinarum*.

In H. influenzae, competence is induced when exponentially growing cells are transferred from rich medium to the starvation medium M-IV (12). In the case of *A. paragallinarum*, we grew cells in tryptic soy broth (TSB) to an optical density at 600 nm (OD_600_) of 0.2, incubated them in M-IV medium for 100 min, and then transformed 1 μg/mL of plasmid pEA-KU or pEA-K. These procedures resulted in abundant resistant colonies. Cells without added DNA did not produce any resistant colonies. Although we have explored a variety of electroporation conditions, no positive transformant was obtained.

### Effects of the DNA concentration and incubation time on natural transformation.

**FIG 2:**
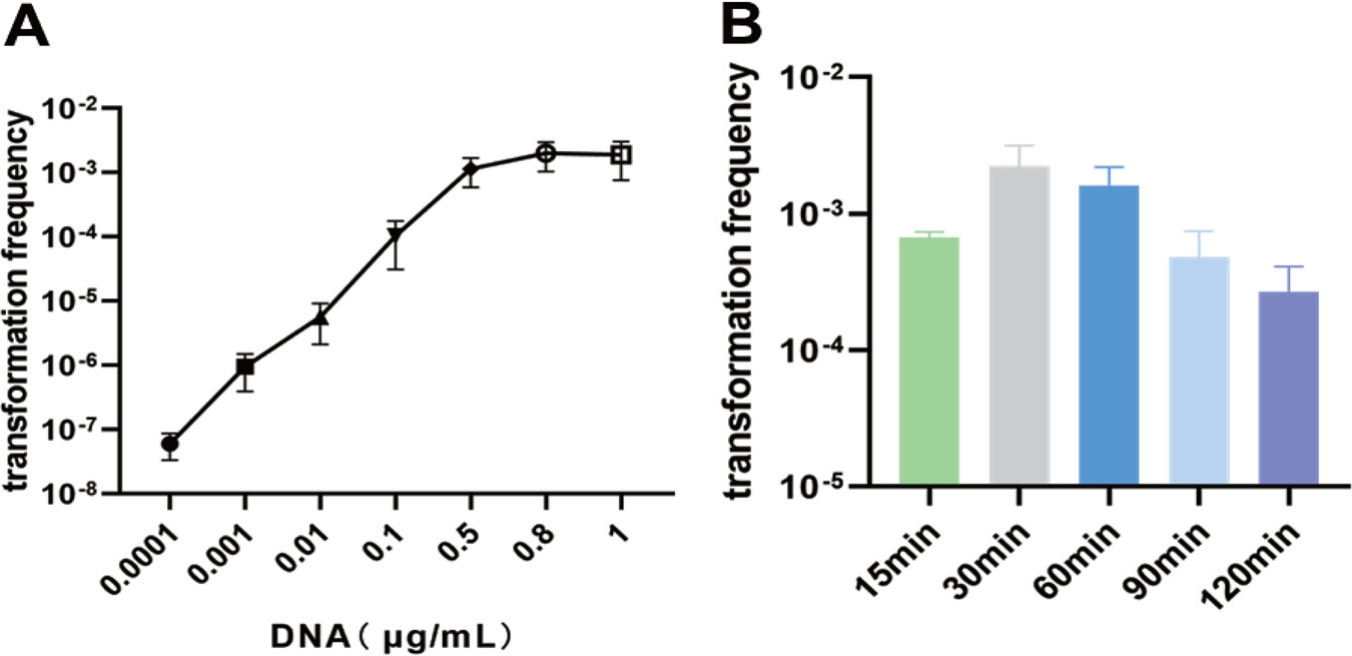
Effect of the DNA concentration or DNA coincubated with competent cells for different times on natural transformation in *A. paragallinarum*. (A) Effect of the DNA concentration on the transformation frequency of *A. paragallinarum*. *A. paragallinarum* 2019/HB64 was transformed with gradient concentrations of pEA-KU. (B) Effect of DNA coincubated with competent cells for different times on the transformation frequency. *A. paragallinarum* 2019/HB64 was coincubated with 1 μg of pEA-KU for the indicated times. The averages and standard deviations from three independent experiments are shown.

To investigate the effect of the DNA concentration on the frequency of natural transformation, we conducted a transformation process on *A. paragallinarum* by varying the concentrations of pEA-KU. Our findings revealed that the transformation frequency increased as the DNA concentration increased within limits. The highest transformation frequency was obtained at saturating DNA concentrations of ≥0.5 μg/mL (Fig. 2A). Furthermore, the transformation frequency was affected by the time of coincubation of 1 μg/mL DNA with competent cells and was highest when the cells were incubated for 30 min (Fig. 2B).

### Natural transformation abilities of different *A. paragallinarum* strains.

Strains 2019/HB64, 2019/HB68, and 2019/NX56 were prepared in competent cells for natural transformation. Briefly, fresh bacterial fluid was added to TSB medium at a ratio of 1:100, cultivation was performed until the OD_600_ reached ~0.2, and the bacteria were collected by centrifugation. The collected cells were resuspended in fresh M-IV medium in equal volumes and incubated at 37°C with shaking (100 rpm) for 100 min. Next, 1 μg/mL plasmid pEA-KU or pEA-K was used for natural transformation. The corresponding transformation frequency was calculated using the plate colony counts after transformation.

**FIG 3:**
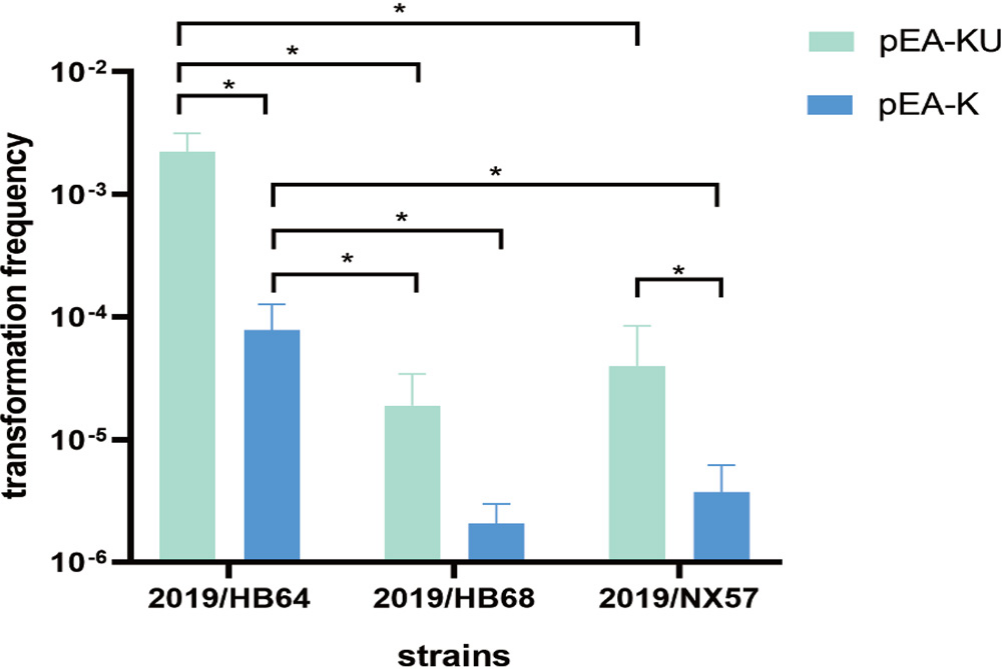
Comparison of transformation frequencies among *A. paragallinarum* strains. * indicates that there was a significant difference between the groups (*P* < 0.05). The averages and standard deviations from three independent experiments are shown.

The results showed that the transformation frequency of the plasmid carrying the USS was higher than that of the plasmid without the USS in all tested strains, which showed that the USS 5′-ACCGCACTT-3′ promoted the uptake of exogenous DNA by *A. paragallinarum* (Fig. 3). In addition, the transformation frequency of plasmid pEA-KU in strain 2019/HB64 was significantly higher than that for strains 2019/HB68 and 2019/NX57 (Fig. 3). The pEA-K plasmid led to a transformation frequency of strain 2019/HB64 that was significantly higher than those of the other strains (Fig. 3). These results indicated that there were significant differences in the frequencies of natural transformation among different strains of *A. paragallinarum*.

## DISCUSSION

In the *Pasteurellaceae*, electroporation is an essential method for the introduction of foreign genes into cells (39, 40), but it is not always effective (41). In this study, we attempted to introduce shuttle plasmids into *A. paragallinarum* by electroporation. Nevertheless, despite exploring various experimental conditions, we could not obtain any transformants. Wang et al. previously found that *A. paragallinarum* strains 221 and H18 could not be successfully transformed by electroporation (3). Similarly, electroporation did not prove successful in introducing the transposons mini-Tn*10* and Tn*5* into *A. paragallinarum* strains TW-1 and 221 (4). This may be due to the obstruction or restriction of structures such as bacterial capsule and the influence of the modification system (42, 43).

Natural transformation is an important mechanism for bacteria to obtain external DNA molecules in the process of evolution (18). It can also be used as a means to introduce foreign genes into bacteria under laboratory conditions. Natural transformation is simpler to perform, is closer to gene transfer under natural conditions, and does not require equipment such as a gene pulser transfection apparatus. In *A. paragallinarum*, 16 competence proteins related to natural competence, DNA uptake, and DNA processing were identified in this study. Some noncompetent bacterial strains have mutations in one or more of the competence genes, which likely explains their failure to transform (17, 21, 44). But in Riemerella anatipestifer, only some homologs of ComE, ComM, DprA, RadC, and Ssb were identified (45). Similarly, we did not find some competence proteins in *A. paragallinarum*, such as ComA, ComB, ComC, and ComD, so it may have a new mechanism of DNA transport.

In order to adapt to various environmental pressures, bacteria have different ways to start the establishment of natural competence to regulate the uptake of foreign genes. For example, Streptococcus pneumoniae forms a natural receptive state under the action of antibiotics such as fluoroquinolones to absorb foreign DNA in the environment (46). The natural competence of Vibrio cholerae can be established by chitosan in the environment, while peptone, phosphate, and iron promote the natural transformation of Riemerella anatipestifer (47). A change in solid-liquid culture conditions can promote the formation of natural competence in H. parasuis (48). When H. influenzae and Bacillus subtilis are transferred from nutrient-rich medium to barren medium, the regulation of their natural transformation competence is activated (12, 49). We found that the competence of *A. paragallinarum* can be induced under the same conditions as those for H. influenzae by transfer to M-IV starvation medium, indicating that their regulations of natural competence share features.

In H. influenzae, homologous DNA fragments (including the USS) hinder the uptake of other fragments by bacteria (50). *Neisseria* is more likely to ingest DNA fragments carrying a USS under the same conditions (51). Bigas et al. found that plasmids without a USS failed to obtain the transformant in the construction of a knockout mutant of H. parasuis that was defective in the *thy* gene (48). Thus, USSs are enriched in the genome over a long period of evolution in bacteria with competence, so one or more USSs appear for every fragment larger than ~2 kb (5, 52).

In H. influenzae Rd, the highly conserved USS is the 9-bp sequence 5′-AAGTGCGGT-3′, which has 1,471 copies in the 1.83-Mb genome (53). The USS of *Neisseriaceae* (5′-GCCGTCTGAA-3′) presents 1,891 copies in the 2.18-Mb genome of Neisseria meningitidis Z2491 (5). In this study, we found that there were 1,537 to 1,641 copies of the sequence 5′-ACCGCACTT-3′ in the genome of *A. paragallinarum*. We compared the transformation frequencies between plasmid pEA-KU carrying the USS (5′-ACCGCACTT-3′) and plasmid pEA-K without the USS in *A. paragallinarum*. The results showed that plasmids carrying the USS were more easily absorbed by *A. paragallinarum*.

There are differences in the efficiencies of natural transformation between different strains, even within the same bacteria. Bossé et al. identified only one highly transformable strain of Actinobacillus pleuropneumoniae among 16 strains (21). The other strains were either nontransformable or poorly transformable. Kristensen et al. tested the natural competence of 9 Gallibacterium anatis strains from different origins (53), among which the transformation frequency of the lowest-transformation-frequency strain, F149T, was almost 3 orders of magnitude lower than that of the highest-transformation-frequency strain, 12656-12.

There were differences in the frequencies of natural transformation among different *A. paragallinarum* strains. We found that the transformation frequency of 2019/HB64 was 10 to 100 times higher than those of 2019/HB68 and 2019/NX57. However, all tested strains could be successfully transformed by natural transformation.

In conclusion, natural competence in *A. paragallinarum* is induced when cells are transferred from a rich medium to a defined starvation medium. In this study, we established a method for transforming a plasmid into naturally competent *A. paragallinarum* strains stably and efficiently. This transformation method could be an important tool for introducing foreign DNA into *A. paragallinarum* and could provide a basis for constructing a genetic operation platform for this species.

## MATERIALS AND METHODS

### Strains and plasmids.

Strains of *A. paragallinarum* were isolated from chickens with clinical signs of IC (39) (Table 5). These strains were grown in TSB (Hopebio, China) or on tryptic soy agar (TSA) (Hopebio, China) supplemented with 10% inactivated bovine serum (Solarbio, China) and 0.0025% (wt/vol) NAD (Sangon Biotech, China). The plates were cultured at 37°C under 5% CO_2_ for 24 to 36 h. Typical colonies were inoculated into TSB and cultured at 37°C in a shaker for 16 h.

**TABLE 5:** *A. paragallinarum* strains and plasmids used in this study

Strain or plasmid	Characteristic	Reference
*A. paragallinarum* strains		
2019/NX57	Serovar C	39
2019/HB65	Serovar A	39
2019/HB68	Serovar B	39
Plasmids		
pEA	pUC57 ori (pYMH5)	This study
pEA-KU	pEA USS::Kan^r^	This study
pEA-K	pEA Kan^r^	This study

**FIG 4:**
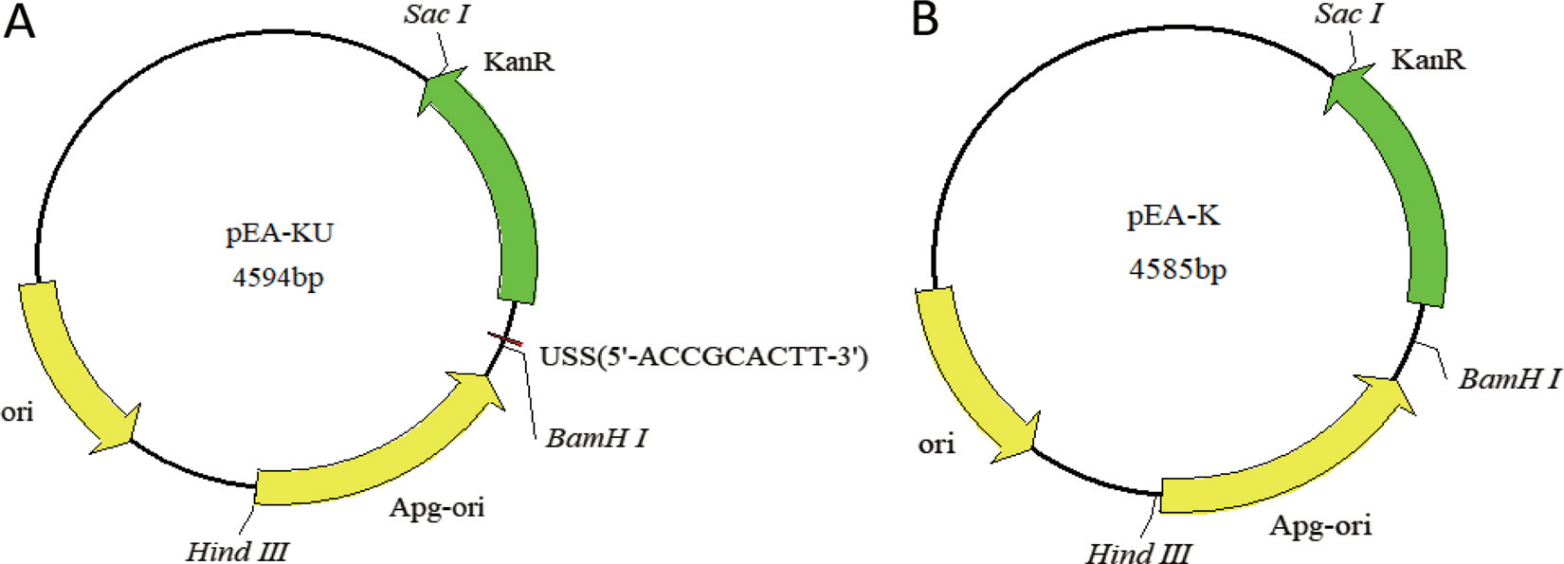
Maps of shuttle plasmids pEA-KU and pEA-K. (A) Map of plasmid pEA-KU. (B) Map of plasmid pEA-K. The region containing the replication origin (Apg-ori) is derived from plasmid pYMH5; the kanamycin resistance (Kan^r^) gene is derived from plasmid pKD4. In pEA-KU, the USS is added at the 5′ end of the Kan^r^ fragment through synthetic primers. The maps were drawn using DNAMan software (version 6.0.3; Lynnon BioSoft).

The replication origin (Apg-ori) (0.9 kb) of the endogenous plasmid pYMH5 (GenBank accession number NC_010912.1) (54) in *A. paragallinarum* was synthesized (General Biol, China). To allow the plasmids to replicate in *A. paragallinarum*, the synthesized Apg-ori was inserted into the HindIII and BamHI sites of pUC57. Next, in order to add a screening marker, the kanamycin resistance (Kan^r^) gene was amplified by PCR from plasmid pKD4 (GenBank accession number AY048743) using primer pair Kan-F1/Kan-R1 (pEA-KU) or Kan-F2/Kan-R2 (pEA-K) (Table 6). The PCR products were restriction digested with BamHI and SacI and inserted into plasmid pUC57 digested with BamHI and SacI to construct the plasmids pEA-KU and pEA-K (Fig. 4). These plasmids were purified from Escherichia coli Trans1-T1 (TransGen, China) with a plasmid minikit (Cwbio, China).

**TABLE 6:** Primers used in this study

Primer	Sequence (5′–3′)^*a*^	Length (bp)
Kan-F1	CGGATCCaccgcacttGCCCTCTGGTAAGGTTGG	1,017
Kan-R1	CGAGCTCAAGGCGGCGGTGGAATCGAAATCTC	

Kan-F2	CGGATCCGCCCTCTGGTAAGGTTGG	1,008
Kan-R2	CGAGCTCAAGGCGGCGGTGGAATCGAAATCTC	

Kan-jd F	ATGATTGAACAAGATGGATTGCACG	874
Kan-jd R	AAGGCGGCGGTGGAATCGAAATCTC	

a The underlined sequence is the enzyme digestion site; lowercase type indicates the USS. A BamHI restriction site was added to Kan-F1 and Kan-F2. A SacI restriction site was added to Kan-R1 and Kan-R2.

### Identification of competence proteins and uptake signal sequences in *A. paragallinarum*.

In natural transformation, competence proteins are involved mainly in gene regulation, DNA uptake, and DNA processing (17, 53, 55). For sequence analysis, the genome sequences of 5 *A. paragallinarum* strains and H. influenzae KW20 were downloaded from GenBank (https://www.ncbi.nlm.nih.gov/genbank/). *A. paragallinarum* strains 2019/JS28, 2019/JS40, and 2019/JS44 were sequenced by our laboratory (56) (Table 7).

**TABLE 7:** Genome information for *A. paragallinarum*

Strain	GenBank accession no.	Origin	Genome size (Mb)
2019/JS28	Unpublished	China	2.80
2019/JS40	Unpublished	China	2.54
2019/JS44	Unpublished	China	2.71
p4chr1	CP081939.1	China	2.77
ADL-AP02	CP051641.1	USA	2.42
AVPG2015	CP058307.1	Mexico	2.53
FARPER-174	CP034110.1	Peru	2.43
ESV-135	CP050316.1	Mexico	2.52

To find and identify competence proteins for natural transformation, the genomes of *A. paragallinarum* were analyzed using the Prokaryotic Genome Annotation Pipeline (PGAP) (version 5.2) (57) and the BLAST function from the NCBI (58). They were subsequently used in a reciprocal search in H. influenzae Rd KW20.

CRP-S sequences were identified by scanning 300 nucleotides (nt) upstream of the start codons of competence transcription units predicted using FIMO software (version 5.4.1) (59). A logo map of the CRP-S locus of *A. paragallinarum* was drawn using WebLogo software (version 2.8.2) (60). The copies of H. influenzae USS cores or H. parasuis USS cores (5, 48) in the *A. paragallinarum* genomes were determined using the search function of SnapGene (version 4.2.4) (http://www.snapgene.com).

### M-IV medium induction of natural competence.

Natural competence was induced by incubation in M-IV medium according to a method described previously by Poje and Redfield (61). Briefly, typical colonies of *A. paragallinarum* were inoculated into TSB and cultured at 37°C in a shaker for 16 h. They were added to TSB medium at a ratio of 1:100, and we continued to cultivate them until the OD_600_ reached ~0.2. The bacteria were collected by centrifugation, and the collected cells were resuspended in fresh M-IV medium in equal volumes, followed by incubation at 37°C with shaking (100 rpm) for 100 min. The cells were then used for transformation or stored in 15% (vol/vol) glycerol at −80°C.

### Procedure for natural transformation.

Before transformation, competent cells were thawed on ice, centrifuged, collected, and resuspended in fresh M-IV medium. Cells (1 mL) were incubated with 1 μg of DNA for 30 min at 37°C. Next, we added 2 mL of TSB, incubated the cells at 37°C for 100 min, serially diluted them, and plated them onto selective plates (usually with kanamycin at 10 μg/mL). A nonselective plate was also coated with the cells as a control. Positive transformants were identified by PCR using primer pair Kan-jd F/Kan-jd R (Table 6). The transformation frequency was calculated as follows: transformation frequency = (number of positive transformants)/(amount of total viable bacteria). The bacteria were cultured for at least 24 h after transformation.

### Electroporation of *A. paragallinarum*.

Exponentially growing cells were diluted 1:100 in TSB medium and incubated at 37°C in a shaker (220 rpm) until the OD_600_ reached 0.6 to 0.8. The culture was incubated on ice for 15 min before electroporation. Cells were collected by centrifugation at 4°C and washed three times in precooled 10% (vol/vol) glycerol. Next, the cells were resuspended in fresh TSB medium (1% of the initial volume).

Suspensions (100 μL) were mixed with 1 μg of DNA and transferred to precooled electroporation cuvettes (gap size of 2 mm). Electroporation was performed using a gene introduction instrument (Scientz, China) with settings of 2.2 kV, 400 Ω, and 25 μF, resulting in time constants of 8 to 10 ms. A total of 1 mL of preheated (37°C) TSB was immediately added, and the cells were incubated for at least 2 h at 37°C. Selective plates containing kanamycin (10 μg/mL) were then coated with the culture. A nonselective plate was also coated with the cells as a control. The transformation frequency was calculated as described above.

### Statistical analysis.

Statistical analysis was performed using GraphPad Prism software (version 6) for Windows. The statistical significance of the data was analyzed using Student’s *t* test. A *P* value of <0.05 was considered significant.
